# Unlocking ferroptosis to overcome cancer stem cells-mediated treatment failure and immune evasion

**DOI:** 10.3389/fimmu.2025.1708850

**Published:** 2026-01-12

**Authors:** Weicheng Jin, Jun Xia, Chuanqiang Zhang, Tongguo Shi, Yu Shen

**Affiliations:** 1 Department of Anesthesiology, Suzhou Ninth Hospital Affiliated to Soochow University, Suzhou, China; 2 Department of Endocrinology, The Affiliated Jiangsu Shengze Hospital of Nanjing Medical University, Suzhou, China; 3 Department of General Surgery, The Affiliated Jiangsu Shengze Hospital of Nanjing Medical University, Suzhou, China; 4 Shengze Clinical Medical College, Kangda College of Nanjing Medical University, Suzhou, China; 5 Jiangsu Institute of Clinical Immunology, The First Affiliated Hospital of Soochow University, Suzhou, China; 6 Department of General Surgery, Suzhou Ninth People’s Hospital, Suzhou Ninth Hospital Affiliated to Soochow University, Suzhou, Jiangsu, China

**Keywords:** biomarker, cancer stem cell, ferroptosis, immune, targeted therapy

## Abstract

Cancer stem cells (CSCs), a small subset of tumor cells with stem cell - like properties, are central to driving tumorigenesis and metastasis. Growing evidence highlights the importance of ferroptosis in maintaining CSCs, positioning ferroptosis as a promising therapeutic strategy. This review explores the interplay between ferroptosis and CSCs, which drive tumor initiation, therapy resistance, and recurrence. It summarizes the mechanisms of ferroptosis involving iron metabolism, lipid peroxidation, and antioxidant defense. The article highlights how non-coding RNAs, metabolic reprogramming, redox homeostasis, and key signaling pathways interconnect ferroptosis with CSC stemness. Furthermore, it discusses the role of ferroptosis in modulating the tumor immune microenvironment and CSC-mediated immune evasion. The identification of stemness and ferroptosis-related biomarkers offers prognostic value, while pharmacological strategies-including traditional Chinese medicine and natural compounds-show promise in targeting CSCs by inducing ferroptosis. Ultimately, leveraging ferroptosis represents a novel therapeutic approach to eliminate therapy-resistant CSCs and improve cancer treatment outcomes.

## 1 Introduction

Tumors are complex systems made up of cancer cells and a tumor microenvironment (TME) that includes cancer-associated fibroblasts, immune cells, blood vessels, and other components (1). Much research has been done on how the TME contributes to tumor heterogeneity, which comes from epigenetic and genetic differences (2). The cancer stem cell (CSC) model suggests that tumors have a cellular hierarchy with CSCs at the top, responsible for maintaining tumorigenicity and the original tumor’s cellular diversity (3, 4). This model has received attention because it clarifies why many treatments that seem to eliminate cancer cells initially often lead to cancer coming back (4). CSCs are often more resistant to current anticancer therapies than the surrounding rapidly dividing bulk tumor cells (5). Various factors, such as genetic mutations, epigenetic changes within tumor cells, and interactions with the TME, all affect CSC behavior and the tumor’s response to therapies, which in turn impacts the overall prognosis (6). Therefore, targeting CSCs within tumors is clinically important to prevent relapse.

Ferroptosis, a non-apoptotic form of cell death, can be inhibited by iron chelators and lipophilic antioxidants (7). Stockwell first reported this in 2012 when Erastin and RSL3 were introduced to RAS-mutated tumor cells (7). Its hallmarks include iron accumulation in the labile iron pool, increased phospholipid peroxidation in PUFA-containing membranes, and insufficient free radical neutralization by antioxidants (8). Studies show ferroptosis is effective against refractory cancer cells in mesenchymal states, persistent drug-resistant cells in residual lesions, and dedifferentiated cancer cells resistant to apoptosis from conventional therapy (9). In recent years, the interplay between ferroptosis and CSCs has emerged as a critical research focus (10, 11). Evidence suggests that iron metabolism is essential for CSC maintenance, making ferroptosis a promising therapeutic approach (12, 13). Ferroptosis inducers can selectively target CSCs within a tumor, suggesting that ferroptosis could be harnessed to induce CSC death (14). Indeed, the high selectivity and efficacy of ferroptosis in inducing CSC death are vital for achieving complete tumor eradication and overcoming chemotherapy resistance (14). CSCs are particularly susceptible to ferroptosis due to their unique metabolic and signaling pathway preferences (15). Understanding ferroptosis’s role in tumor CSCs could provide valuable insights for developing therapeutic strategies against tumors.

This review presents an overview of the mechanisms underlying ferroptosis and the contributions of CSCs to tumorigenesis. An analysis of the interplay between ferroptosis and cellular stemness follows, discussing its potential therapeutic implications. Furthermore, an examination is provided on how ferroptosis influences the tumor immune microenvironment and facilitates immune evasion by CSCs. The article concludes by summarizing biomarkers linked to both stemness and ferroptosis, alongside pharmacological approaches designed to induce ferroptosis in CSCs.

## 2 An overview of ferroptosis

**Figure 1:**
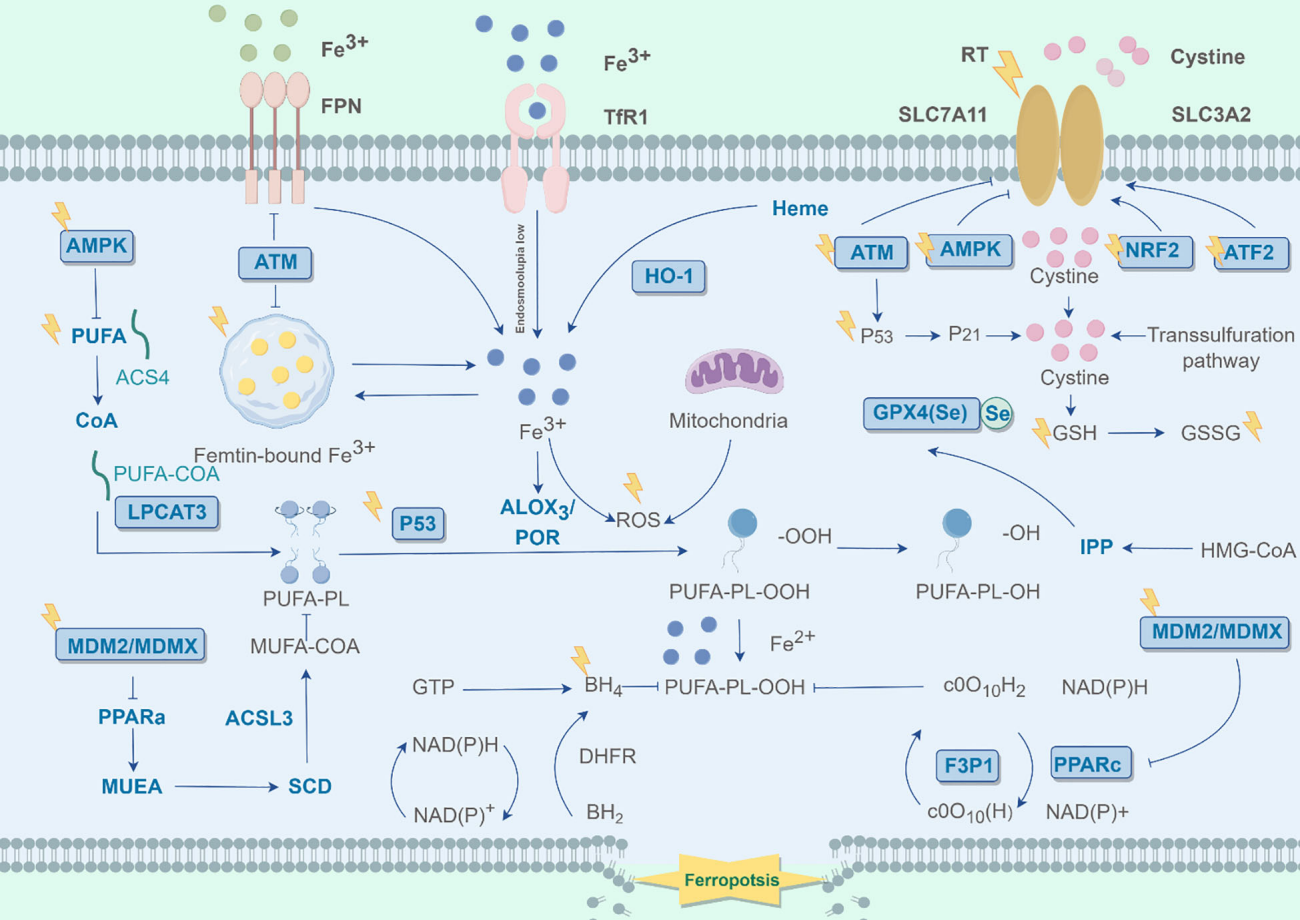
Mechanisms of ferroptosis in cells. The graphic was created by Figdraw (www.figdraw.com).

Ferroptosis is a novel non-apoptotic form of programmed cell death, marked by iron-dependent lipid ROS accumulation, distinct from apoptosis, necrosis, and autophagy. It is driven by intracellular iron accumulation, glutathione (GSH) depletion, and lipid peroxidation in a positive feedback loop (**Figure 1**) (7).

Iron metabolism comprises uptake, distribution, storage, utilization, and efflux, maintaining a dynamic intracellular balance (16). Disruptions in this balance can directly or indirectly alter cellular susceptibility to death, particularly ferroptosis. Cells import iron through transferrin-bound ferric ions (Fe³^+^). These are reduced to ferrous ions (Fe²^+^) by endosomal ferric reductases and transported to the cytosol via divalent metal transporter 1. Transferrin receptor 1 (TfR1) mediates the cellular uptake of iron from the extracellular environment, thereby supplying the iron necessary for ferroptosis. The upregulation of TfR1 expression enhances this iron import, leading to the accumulation of intracellular labile iron and promoting ferroptosis execution (17). The iron transport/export system, primarily consisting of ferroportin (FPN), cytosolic iron chaperones (e.g., PCBP1 and PCBP2), and heme transporters, functions to export iron from the cell and distribute it intracellularly. It directly determines ferroptosis susceptibility by governing the “efflux” and “distribution” of intracellular iron ions (18).

Fe²^+^ can then be oxidized back to Fe³^+^ via the Fenton reaction, which drives lipid peroxidation and generates reactive oxygen species (ROS) (19). Ferritin, a heteropolymer comprising ferritin light chain (FTL) and ferritin heavy chain 1 (FTH1), sequesters Fe³^+^ that has not undergone reduction to Fe²^+^ by DMT1. This process directly diminishes the intracellular pool of labile iron ions, which act as central catalysts for lipid peroxidation and subsequent ferroptosis induction. Consequently, downregulation of either FTH1 or FTL expression compromises the capacity or functionality of this protective iron storage system, resulting in the accumulation of labile iron and heightened cellular susceptibility to ferroptosis (19, 20).

Several regulatory proteins and receptors, along with other proteins, can influence ferroptosis by altering lipid peroxidation and mitochondrial function through iron metabolism control (21). Heat shock protein family B member 1 (HSPB1) can inhibit TFR1 expression and lower intracellular iron levels, and its overexpression significantly suppresses ferroptosis (22). Iron response element binding protein 2 (IREB2) boosts FTL and FTH1 expression, thereby inhibiting erastin-induced ferroptosis (23). Nuclear factor erythroid 2-related factor 2 (NRF2), an antioxidant response regulator, plays a dual role in regulating ferroptosis. It upregulates the expression of ferritin heavy chain (FTH1) and ferritin light chain (FTL), thereby promoting iron storage protein expression, suppressing ferritinophagy, and reducing intracellular labile iron levels (24). By enhancing the functionality of the GSH-GPX4 axis, NRF2 effectively inhibits lipid peroxidation (25).

Ferroptosis regulation also involves the amino acid metabolism pathway, particularly the system Xc--GSH-GPX4 axis (26). System Xc-, an amino acid transporter in phospholipid bilayer membranes, is formed by solute carrier family 7 member 11 (SLC7A11) and solute carrier family 3 member 2 (SLC3A2) (27). It primarily facilitates the exchange of intracellular glutamate for extracellular cystine, providing a substrate for GSH synthesis, a key component of the antioxidant system (28). Inhibiting system Xc- activity reduces cystine uptake, disrupts GSH synthesis, and weakens antioxidant capacity, leading to lipid ROS accumulation, oxidative damage, and ferroptosis (26).

Lipid peroxidation, a key driver of ferroptosis, involves both enzymatic and non-enzymatic pathways (29). Enzymatically, lipoxygenases (ALOXs), non-heme iron enzymes, catalyze the peroxidation of polyunsaturated fatty acids (PUFAs) in membrane phospholipids, generating cytotoxic lipid hydroperoxides (30). This process enhances membrane fluidity and permeability, impairing membrane function and triggering ferroptosis. Non-enzymatically, the Fenton reaction, driven by labile iron, also generates ROS that promote lipid peroxidation (21).

The susceptibility to lipid peroxidation is fundamentally determined by the composition of membrane phospholipids, which is shaped by specific lipid-metabolizing enzymes (31). Acyl-CoA synthetase long-chain family member 4 (ACSL4) acts as a critical determinant of ferroptosis sensitivity by selectively activating PUFAs and facilitating their incorporation into membrane phospholipids, thereby supplying the essential substrates for peroxidation (32). These integrated PUFA-containing phospholipids (PL-PUFAs) subsequently undergo oxidation in iron-dependent reactions, catalyzed either by reactive oxygen species from the Fenton reaction or by enzymes such as ALOX15, leading to massive production of lipid hydroperoxides (PL-PUFA-OOH) (33). Consequently, elevated ACSL4 expression in certain cancer cells renders them exceptionally vulnerable to ferroptosis inducers (34).

Working in concert with ACSL4, lysophosphatidylcholine acyltransferase 3 (LPCAT3) executes the subsequent crucial step by specifically esterifying these activated PUFAs into phosphatidethanolamine (PE) at the sn-2 position, generating PUFA-PE (35). This reaction is indispensable for establishing a pro-ferroptotic membrane landscape (21). LPCAT3 expression strongly correlates with ferroptosis susceptibility, and its high expression in cancer cells directly predicts hypersensitivity to GPX4 inhibitors (36). When GPX4-mediated defense is compromised, the LPCAT3-generated PUFA-PE membrane pool becomes the primary site for extensive lipid peroxidation, irreversibly driving ferroptosis (36, 37).

Beyond the canonical PUFA-phospholipid pathway, other lipid species significantly modulate ferroptotic sensitivity (38). Ether lipids, particularly plasmalogens, represent abundant membrane components that possess a unique vinyl ether bond at the sn-1 position (38, 39). This chemical structure exhibits heightened susceptibility to oxidative attack compared to conventional ester bonds. Upon radical initiation, this bond not only undergoes oxidation but also efficiently propagates and amplifies the lipid peroxidation chain reaction, thereby accelerating membrane damage dissemination (38, 39).

In contrast, monounsaturated fatty acids (MUFAs) exert potent anti-ferroptotic effects through multiple mechanisms (40). They incorporate into membrane phospholipids via ACSL3-mediated esterification, effectively diluting the density of peroxidation-sensitive PUFAs. Additionally, MUFAs competitively inhibit ACSL4-driven PUFA activation. Furthermore, MUFA derivatives such as oleoylethanolamide may indirectly bolster cellular antioxidant defenses by activating transcription factors like NF-κB or Nrf2, leading to upregulation of cytoprotective genes (40, 41). Ultimately, MUFA supplementation restricts oxidative membrane damage and reduces pro-ferroptotic phospholipid substrates, significantly enhancing cellular resistance (42). Stearoyl-CoA Desaturase-1 (SCD1), the key rate-limiting enzyme in MUFA biosynthesis, critically determines ferroptosis sensitivity through its role in membrane lipid remodeling. Typically, SCD1 upregulation or activation elevates MUFA abundance, leading to a decreased PUFA/MUFA ratio in membrane phospholipids and a consequent significant reduction in ferroptosis susceptibility, thereby enhancing cellular defense (43). For example, chemotoxicity-induced exosomal lncFERO regulates ferroptosis and stemness in gastric cancer stem cells through the hnRNPA1/SCD1 axis (44). Collectively, these insights underscore the paramount importance of lipid metabolic networks in determining cellular fate through ferroptosis, revealing complex regulatory interactions between pro- and anti-ferroptotic lipid species.

Recently, researchers put forward the view that autophagy plays a complex and dual role in ferroptosis, as it can both inhibit and promote the occurrence of ferroptosis (45). On one hand, autophagy can promote ferroptosis by degrading iron - storage proteins (such as ferritin) and releasing iron ions, thereby increasing the available intracellular iron content (46). For instance, under certain pathological conditions, autophagy - related proteins (such as NCOA4) - mediated ferritinophagy is enhanced. This leads to an increase in released ions, exacerbates lipid peroxidation and cell damage, and thus accelerates the process of ferroptosis (47). On the other hand, autophagy can also inhibit ferroptosis by clearing damaged organelles and harmful substances, thereby alleviating intracellular oxidative stress and iron overload (48).

In summary, ferroptosis is a complex form of cell death whose mechanism involves iron metabolism, lipid peroxidation, and the regulation of various signaling pathways. A deeper understanding of the mechanisms of ferroptosis can not only help to understand its role in diseases, but may also offer new targets and strategies for the treatment of related diseases.

## 3 CSCs: roles in tumor progression, drug resistance, and immune evasion

**Figure 2:**
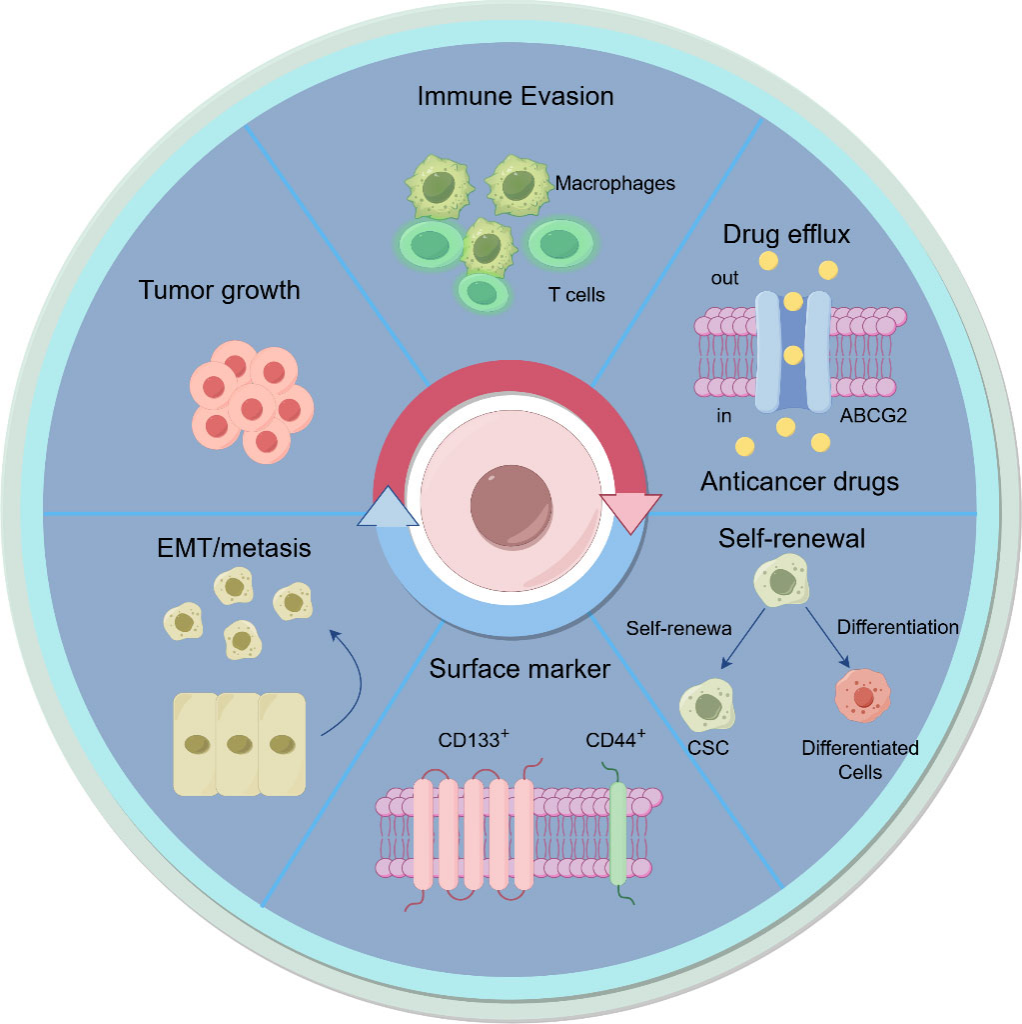
The biological functions of CSCs in cancer. CSCs plays crucial roles in tumor growth, metastasis, drug resistance, and immune evasion, highlighting their impact on tumor progression. The graphic was created using Figdraw (www.figdraw.com).

CSCs, a small subset of tumor cells with stem cell - like properties such as - renewal, multi-lineage differentiation, and unlimited proliferation, are considered the root of tumorigenesis, development, recurrence, and metastasis (3, 49). Up to now, a variety of markers have been utilized to identify CSCs across different types of cancer, such as CD24, CD44, CD133, ALDH, ABCB5, EpCAM, LGR5, and so on. These CSCs possess the remarkable ability to proliferate extensively and renew themselves, allowing them to initiate tumors in both hosts with compromised immune systems and those with fully functional immune systems (3, 49).CSCs in tumors are thought to play a crucial role in tumor formation, resistance to therapy, immune evasion and have the potential to induce metastasis via the epithelial-mesenchymal transition (EMT) process (**Figure 2**).

CSCs play a central role in tumor progression by maintaining tumor heterogeneity and promoting tumor growth and metastasis. They influence tumor progression through multiple signaling pathways and interactions with the TME (50). For example, IL-6 secreted by tumor-associated macrophages (TAMs) can activate the JAK2/STAT3 pathway, up-regulate xCT expression, induce ferroptosis resistance, and promote the progression of head and neck squamous cell carcinoma (51). Breast CSCs secrete DKK1 to promote the differentiation of metastatic cancer cells and enhance their ferroptosis resistance, favoring metastatic niche formation (52). Moreover, exosomes secreted by CSCs can carry bioactive molecules like proteins and miRNAs, modulating the TME to facilitate cancer cell invasion and metastasis (53).

CSCs contribute to drug resistance and cancer relapse (54). They exhibit high activity of drug efflux pumps such as ABCG2, which actively expel chemotherapeutic drugs from the cell, reducing intracellular drug concentrations (55). They also possess efficient DNA damage repair mechanisms, effectively repairing DNA damage caused by chemotherapeutic drugs or radiation (56). CSCs often remain in a relatively quiescent G0 phase of the cell cycle, making them less sensitive to chemotherapeutic drugs that primarily target actively proliferating cells (57). Additionally, certain signaling pathways like PI3K/AKT/mTOR and Notch are involved in CSC-mediated drug resistance (58).

CSCs can evade immune destruction through various mechanisms (59). They up-regulate the expression of immune checkpoint ligands such as PD - L1, which binds to PD-1 on T-cell surfaces, inducing T-cell exhaustion (60). CSCs often have low expression of major histocompatibility complex class I (MHC-I) and other antigen - presenting molecules, reducing the ability of immune cells to recognize and target them (61). They can secrete various cytokines and chemokines to recruit immunosuppressive cells like Tregs and MDSCs, creating an immunosuppressive TME (62). Furthermore, dysregulated signaling pathways such as Wnt/β-catenin in CSCs can enhance their ability to evade immune attacks (60).

In conclusion, CSCs play significant roles in tumor progression, drug resistance, and immune evasion. Targeting CSCs, along with their associated signaling pathways and microenvironment interactions, is a promising strategy for inhibiting tumor progression and metastasis. Understanding the immune evasion mechanisms of CSCs is crucial for developing effective immunotherapies. Future research should focus on further exploring these mechanisms and developing targeted therapies to improve cancer treatment outcomes.

## 4 Targeting CSCs through ferroptosis: molecular mechanisms and therapeutic perspectives

In recent years, ferroptosis has emerged as a critical factor in CSC research. Evidence suggests that iron metabolism is essential for CSC maintenance. The high iron levels in CSCs, associated with their iron dependency, position ferroptosis as a promising approach for CSC- targeted therapy (52, 63). A deeper understanding of ferroptosis mechanisms and its role in CSCs is key to developing more effective cancer treatments and enhancing patient outcomes. In this context, we provide an overview of the relationship between ferroptosis and stemness in cancer cells.

### 4.1 Non-coding RNAs serve as mediators connecting ferroptosis and cancer cell stemness

Non-coding RNAs (ncRNAs), including miRNAs, long noncoding RNAs (lncRNAs), and circular RNAs (circRNAs), are RNA molecules not translated into proteins (64). They are diverse and crucial in various biological processes, such as ferroptosis and cancer cell stemness (**Table 1**).

**Table 1:** NcRNAs mediate the relationship between ferroptosis and stemness in cancers.

ncRNAs	Cancer type	Target	Pathway	Ref.
miR-375	GC	SLC7A11	/	(65)
miR-485-3p	PDAC	SLC7A11	/	(66)
LINC01606	CRC	/	SCD1/Wnt/β-catenin/TFE3	(67)
MIR4435-2HG	Pancreatic cancer	/	miR-1252-5p/STAT1	(68)
LncFERO	GC	/	hnRNPA1/SCD1	(44)
CircRPPH1	GC	/	miR-375/SLC7A11	(69)
CircVPS8	glioma	/	scaffolding MKRN1, SOX15, HNF4A	(70)
circLRFN5	glioma	/	hnRNPA1/SCD1	(71)

MiRNAs are short endogenous, evolutionarily conserved single-stranded RNA molecules, approximately 19–25 nucleotides in length, and are widely associated with CSC-like traits and ferroptosis (72). For example, miR-375, a tumor suppressor in various tumors, suppresses gastric cancer (GC) stemness by inducing SLC7A11-dependent ferroptosis (65). Additionally, miR-485-3p, upregulated via hypoxia-induced epigenetic regulation, enhances pancreatic ductal adenocarcinoma (PDAC) cell stemness and gemcitabine resistance through SLC7A11-mediated ferroptosis (66).

LncRNAs, defined as transcripts exceeding 200 nucleotides, have recently gained attention for their roles in tumour hallmarks, including migration, apoptosis, stemness, drug resistance, and ferroptosis (73). For instance, lncRNA LINC01606, which is upregulated in colorectal cancer (CRC) and linked to poor prognosis, promotes CRC cell proliferation, invasion, and CSC-like characteristics. It also protects CRC cells from ferroptotic cell death via SCD1-Wnt/β-catenin-TFE3 feedback loop signalling (67). Xie et al. reported that lncRNA MIR4435-2HG overexpression enhances pancreatic CSC self-renewal and tumorigenicity by sponging miR-1252-5p to upregulate STAT1. Furthermore, depleting MIR4435-2HG increases pancreatic cancer cells’ sensitivity to gemcitabine-induced growth inhibition and ferroptosis (68). Additionally, exo-lncFERO from GC cells alleviates ferroptosis, thereby regulating the tumorigenic traits of gastric CSCs through the hnRNPA1/SCD1 axis (44).

CircRNAs, a distinct class of ncRNAs, form covalently closed loops without 5’ caps and 3’ poly(A) tails. Dysregulated circRNA expression significantly impact tumour progression, metastasis, stemness, and ferroptosis (74). CircRPPH1, elevated in GC cell spheres and correlates positively with CSC marker expression, sponges miR-375 to upregulate SLC7A11, thereby regulating ferroptosis (69). CircVPS8 enhances glioma CSC viability, proliferation, and stemness, and suppresses ferroptosis by scaffolding MKRN1, SOX15, and HNF4A (70). Conversely, circLRFN5 overexpression reduces glioma CSC viability, proliferation, and tumorigenicity by inducing PRRX2/GCH1 - mediated ferroptosis (71).

Overall, ncRNAs are critical regulators of ferroptosis and cancer cell stemness across various cancer types. Future research into ncRNAs’ roles in these areas holds great promise. Investigating their complex mechanisms can enhance our understanding of ferroptosis and cancer cell stemness. Moreover, targeting ncRNAs may provide new cancer - fighting strategies. By modulating ferroptosis and CSC-related pathways, these approaches could potentially improve treatment outcomes and boost patient survival rates.

### 4.2 Metabolic reprogramming modulates ferroptosis and cancer cell stemness

Cancer cells undergo metabolic reprogramming to support rapid growth and survival, altering pathways like glycolysis, glutaminolysis, and lipid metabolism to meet their bioenergetic and biosynthetic needs (75). This reprogramming, a recognized cancer hallmark, is closely tied to tumor biology aspects such as cancer cell stemness and ferroptosis (75). For instance, dichloroacetate (DCA), a metabolic regulator used for lactic acidosis and mitochondrial disorders, induces ferroptosis in CRC cells. It increases iron and lipid peroxide levels, elevates GSH, and reduces cell viability, thereby curbing CRC cell stemness (76). Similarly, the C2–4 inhibitor, which blocks protein kinase C α (PKCα), sensitizes neuroblastoma CSCs to etoposide. It reduces intracellular GSH levels, shifts metabolism from OXPHOS to aerobic glycolysis, downregulates GPX4 activity, promotes lipid peroxidation, and triggers ferroptosis (77). In hepatocellular carcinoma (HCC), IFN-γ-induced PD-L1 mitochondrial translocation enhances glycolytic reprogramming. This mechanism boosts GPX4-dependent ferroptosis resistance and cancer stemness in liver CSCs (78). Besides, SETD5, a non-typical histone lysine methyltransferase linked to cancer stemness, predicts poor survival in NSCLC. It promotes glycolysis, inhibits ferroptosis, and supports NSCLC stemness, driving tumor metastasis and poor prognosis. Notably, SETD5 achieves these effects through m_6_A-mediated stabilization of PKM2 (79). Meanwhile, in esophageal squamous cell carcinoma (ESCC), the CDK7-YAP-LDHD axis helps ESCC-CSCs evade D-lactate-induced ferroptosis and generates pyruvate to meet their energy needs for self-renewal (10). Moreover, manipulating stearoyl-CoA desaturase (SCD1) via knockdown or cholesterol intake regulation significantly impacts gastric CSC stemness and ferroptosis susceptibility through the SQLE/cholesterol/mTOR pathway (80). Additionally, DRP1 knockdown-induced imbalanced mitochondrial dynamics in oral squamous cell carcinoma cells suppress stemness and drive ferroptosis by enhancing glutaminolysis via the ASCT2/SNAI2 axis (81). Furthermore, Frizzled-7 marks platinum-tolerant ovarian cancer cells with stemness features and altered GSH metabolism. These cells rely on GPX4 for survival and are highly vulnerable to ferroptosis (82).

**Figure 3:**
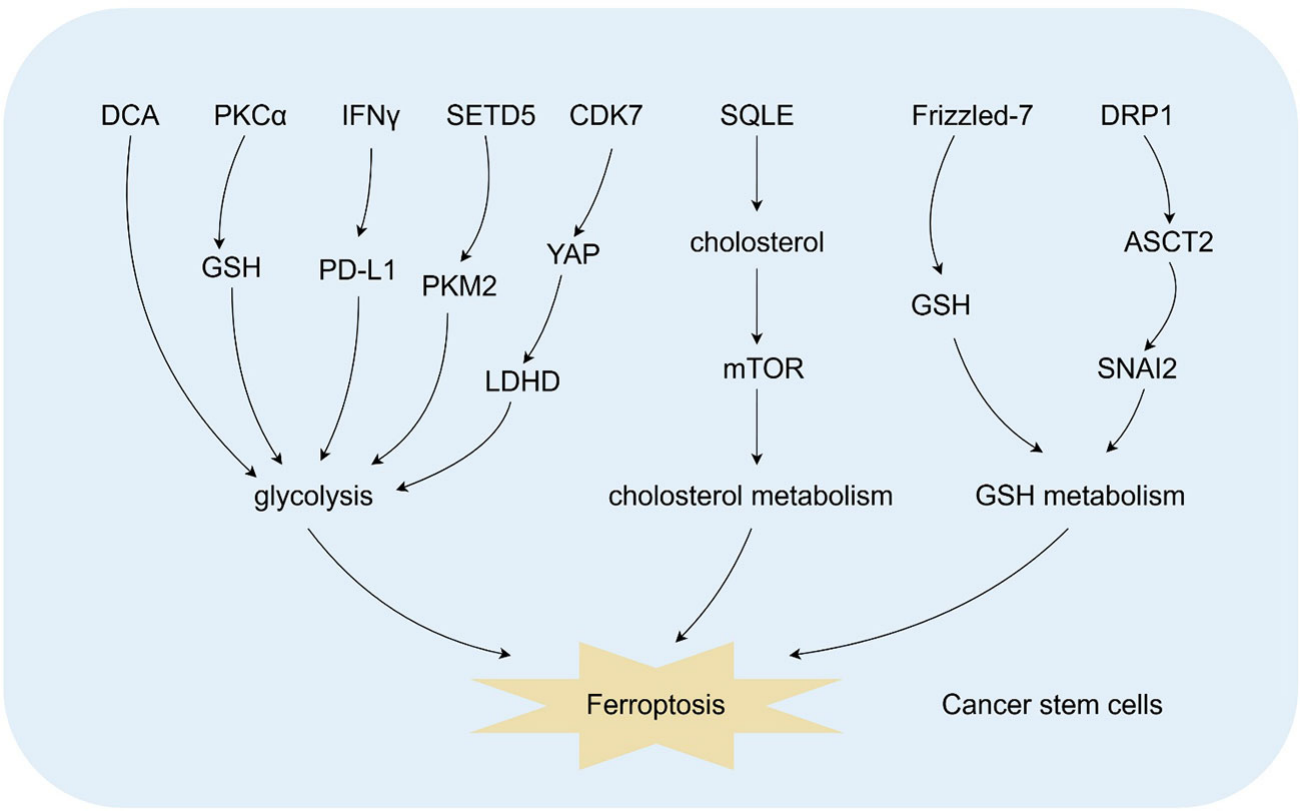
Metabolic reprogramming modulates ferroptosis in CSCs. Cancer metabolic reprogramming, including glycolysis, glutaminolysis, and lipid metabolism, regulats ferroptosis in CSCs. The graphic was created using Figdraw (www.figdraw.com).

In summary, cancer metabolic reprogramming, including glycolysis, glutaminolysis, and lipid metabolism, is a key characteristic that connects to cancer cell stemness and sensitivity to ferroptosis (**Figure 3**). The research emphasizes the therapeutic potential of targeting metabolic pathways to disrupt cancer stemness and trigger ferroptosis. Developing well - designed combinations of metabolic or ferroptosis inducers with standard therapies or other targeted agents shows great promise for creating innovative and effective therapies aimed at eliminating therapy-resistant CSCs. 

### 4.3 Redox homeostasis is critical regulator of ferroptosis and cancer cell stemness

Oxidative-reductive homeostasis is a critical regulator of ferroptosis and CSCs maintenance (83, 84). CSCs rely on robust antioxidant systems to maintain low ROS levels, thereby preserving their stemness (83). Zinc finger MYND-type containing 8 (ZMYND8) protects breast CSCs from oxidative stress and ferroptosis by activating the NRF2 pathway (85). Temozolomide (TMZ)-resistant glioblastoma multiforme (GBM) cells exhibit mesenchymal and stemness traits and resist erastin-induced ferroptosis by activating the CYBB/NRF2/SOD2 pathway (86).

Sulfasalazine, an xCT inhibitor repurposed as an antineoplastic, induces ferroptosis and oxidative stress in oral and breast CSCs by inhibiting GPx-4, a key enzyme that neutralizes oxidative stress products (87). In acute myeloid leukemia (AML), GADD45A, a stress-responsive downstream effector of the LGR4 pathway, plays a protective role by restraining leukemia-initiating activity and enhancing oxidative defense. Its deletion promotes leukemia stem cell (LSC) self-renewal and stemness, and confers ferroptosis resistance through aberrant activation of antioxidant pathways involved in iron and ROS detoxification (88). Further underscoring the metabolic plasticity of CSCs, Wang et al. demonstrated that in oral squamous cell carcinoma (OSCC), inhibition of DRP1 induces mitochondrial elongation, which suppresses cancer stemness and promotes glutaminolysis, thereby driving ferroptosis (81). Similarly, in ovarian cancer stem-like cells (OCSLCs), elevated levels of frataxin (FXN) correlate with increased ferroptosis susceptibility. FXN knockdown reduces the expression of stemness markers such as Nanog, elevates ROS production, and impairs cell viability via PRDX3-mediated oxidative stress (89). Conversely, Ascenzi et al. revealed that lung adenocarcinoma CSCs under 3D stem-like conditions upregulate antioxidant genes and proteins involved in iron storage and export, creating a molecular profile that limits iron availability and protects against ferroptosis initiation (90).

Collectively, these findings underscore the complex interplay between redox balance, ferroptosis, and CSC maintenance. This intricate relationship reveals that CSCs critically depend on robust antioxidant systems to preserve stemness and evade ferroptotic death. Importantly, such redox dependencies represent promising therapeutic vulnerabilities. Targeting these pathways holds significant potential to eradicate therapy-resistant CSC populations and ultimately improve cancer treatment outcomes.

### 4.4 Signaling pathways modulate of ferroptosis and cancer cell stemness

Multiple signaling pathways, including the β-catenin pathway, modulate ferroptosis in CSCs. Normally, β-catenin is phosphorylated by a destruction complex and marked for degradation. However, in many cancers, this pathway is dysregulated, leading to β-catenin accumulation in the cytoplasm and its translocation to the nucleus. There, it acts as a transcriptional co-activator, influencing genes related to cell proliferation, stemness, and survival (91). β-catenin, in conjunction with T-cell factor/lymphoid enhancing factor (TCF/LEF) proteins, plays a crucial role in the Wnt signaling cascade (92). Lv et al. identified IFNγ as a key cytokine that arrests intestinal stem cells through the IFNγ/IFNGR2/APC/TCF4/GPX4 axis. They demonstrated that IFNγ induces GPX4-dependent ferroptosis to eliminate CRC stem cells and showed its synergistic anticancer effect with cold atmospheric plasma in killing CRC cells (93). Another study revealed that USP8 depletion reduces HCC cell proliferation, invasion, and stemness while inducing ferroptosis, effects that can be reversed by β-catenin overexpression. Furthermore, the USP8 inhibitor DUB-IN-3 suppresses aggressive HCC phenotypes and triggers ferroptosis by degrading β-catenin (94). These findings underscore the potential of targeting the β-catenin pathway as a promising strategy for cancer treatment basd on ferroptosis and cancer cell stemness.

Overall, we systematically elaborates on the molecular mechanisms and prospects of the emerging therapeutic strategy of targeting and eliminating CSCs via ferroptosis induction. The core idea is that to maintain their stemness and survival, CSCs exhibit unique iron metabolism dependence and an active antioxidant system. This ironically makes them both sensitive and vulnerable to ferroptosis. Specifically, various molecular mechanisms precisely regulate this process: Non-coding RNAs (e.g., miR - 375, LINC01606, CircRPPH1) act as key mediators, influencing CSC stemness and ferroptosis sensitivity by regulating targets like SLC7A11; metabolic reprogramming (including glycolysis, glutaminolysis, and lipid metabolism) reshapes the redox state within CSCs, directly determining their ferroptosis susceptibility; targeting antioxidant pathways like NRF2 can disrupt CSC defense systems and induce ferroptosis; and key signaling pathways (e.g., Wnt/β - catenin) interact with ferroptosis, offering new intervention targets.

## 5 Ferroptosis mediated the interaction between CSCs and immune response

Recent research indicates that the TME, as a component of the CSC niche, is pivotal for CSC viability and stemness preservation (3, 59). CSCs emit inflammatory mediators that attract and prompt immunosuppressive cells, thereby fostering an immunosuppressive condition within the TME (3, 59). The interplay between CSCs and the TME exerts a decisive influence on the destiny of CSCs, suggesting that solely targeting stem cell signaling pathways may be inadequate to eliminate CSCs. As such, the key proteins involved in the dialogue between CSCs and the TME merit deeper investigation as prospective therapeutic targets for future cancer treatments. Notably, ferroptosis holds a vital position in the interaction between CSCs and the TME (95). Herein, we discussed the roles of ferroptosis in regulating the interaction between CSCs and the TME in cancers.

### 5.1 Ferroptosis-related genes and their correlation with CSCs and immune response

Ferroptosis offers a novel approach to selectively target these cells, reducing their tumor-initiating capacity and increasing their sensitivity to immune surveillance. For instance, the ferroptosis-related gene TXNIP has been shown to play a crucial role in mediating ferroptosis in lung CSCs. This gene’s expression correlates with immunosuppressive microenvironments and histological differentiation, highlighting its importance in the crosstalk between CSCs and the immune response (96). Du et al. identified a collection of ferroptosis-related stemness genes (FRSG) in HCC and demonstrated their potential role in immune infiltration. Using Cox regression analysis, a prognostic risk model was established based on four FRSGs—CDKN2A, GABARAPL1, HRAS, and RPL8. This model holds predictive value for prognosis in HCC, underscoring the importance of these genes in the interaction between CSCs and the immune response (97). Cross-platform dataset analysis identified two stable gastric cancer (GC) stem cell subtypes: low stem cell enrichment (SCE_L) and high stem cell enrichment (SCE_H). Gene set enrichment analysis revealed that ferroptosis, NK cell activation, and post-mutation repair pathways were active in the SCE_L subtype. This finding suggests that ferroptosis-related pathways are closely linked to the immune response in CSCs, potentially influencing their behavior and therapeutic response (98).

The identification and characterization of ferroptosis-related genes have significantly advanced our understanding of the interaction between CSCs and the immune response. These genes not only mediate ferroptosis in CSCs but also influence immune infiltration and overall tumor behavior. Future research should focus on elucidating the precise mechanisms by which these genes modulate the crosstalk between CSCs and the immune microenvironment, paving the way for novel therapeutic strategies that harness ferroptosis to enhance antitumor immunity.

### 5.2 The role of ferroptosis in the immune evasion mechanisms of CSCs

Ferroptosis not only play a significant role in the survival and drug resistance of CSCs but are also closely related to immune evasion mechanisms (12, 99). CPT1A is a key enzyme that regulates the rate of fatty acid oxidation. In lung cancer, CPT1A collaborates with L-carnitine, which is produced by TAMs, to confer resistance to ferroptosis in CSCs and inactivate CD8+ T cells. Mechanistically, CPT1A inhibits the ubiquitination and degradation of c-Myc, while c-Myc, in turn, upregulates the expression of CPT1A. This positive feedback loop between CPT1A and c-Myc significantly enhances the cellular antioxidant capacity by activating the NRF2/GPX4 pathway and reduces the levels of phospholipid polyunsaturated fatty acids through the downregulation of ACSL4, thereby thwarting ferroptosis in CSCs. Importantly, targeting CPT1A can enhance immune checkpoint blockade-induced anti-tumor immunity and augment tumoral ferroptosis in tumor-bearing mice (100). In breast cancer stem cells, the protein levels of Cd274 and Tnfaip3 were found to be upregulated following treatment with salinomycin. In 4T1 tumors treated with cyclophosphamide, the single-cell expression of Cd274 increased in both myeloid- and lymphoid-infiltrated cells, regardless of its receptor Pdcd1 (101). This suggests that CSCs may evade immune surveillance by upregulating immune checkpoint-related proteins. FTO inhibitor-loaded GSH-bioimprinted nanocomposites (GNPIPP12MA) have been developed to enhance anti-leukemogenesis by targeting the FTO/m6A pathway and depleting GSH. GNPIPP12MA can selectively target leukemia cells, particularly leukemia stem cells (LSCs), and trigger ferroptosis by disrupting the intracellular redox balance. Moreover, GNPIPP12MA enhances the efficacy of PD-L1 blockade by boosting the infiltration of cytotoxic T cells, thereby bolstering anti-leukemia immunity (102).

In sum, ferroptosis plays a significant role in the immune evasion of CSCs. These findings provide a theoretical basis for developing new therapeutic strategies that can enhance anti-tumor immune responses by targeting ferroptosis-related genes.

### 5.3 Targeted ferroptosis-immune therapies for cancer stem cells

The interplay between CSCs and immune cells has become a focal point in cancer research (103) , with the goal of developing therapies that can effectively target and eliminate CSCs while enhancing immune responses. Recent advancements in nanotechnology and targeted drug delivery have paved the way for innovative approaches that combine ferroptosis induction with immunotherapy to achieve this goal.

Zhao et al. have developed a nanointegrative approach to achieve CSC-targeted ferroptosis-immunotherapy through the spatiotemporally controlled reprogramming of STAT3-regulated signaling circuits. Specifically, they incorporated the STAT3 inhibitor niclosamide (Ni) and the experimental ferroptosis drug (1S, 3R)-RSL3 (RSL3) into hyaluronic acid-modified amorphous calcium phosphate (ACP) nanounits (CaP-PEG-HA@Ni/RSL3). These nanounits, which can be recognized by CD44-overexpressing CSCs, are released in a coordinated manner. Niclosamide suppresses the CSC-intrinsic STAT3-PD-L1 axis, thereby invigorating adaptive immunity and augmenting IFNγ secretion by CD8+ T cells (104). In a similar vein, an aerosolized nanocatalytic medicinal approach using iron-based nanoparticles has been developed to modulate TAMs and precisely target CSC niches during the nascent phase of lung cancer. These nanoparticles can efficiently accumulate within pulmonary microlesions via dextran-mediated TAM targeting through aerosol administration. Once internalized by TAMs, these nanoparticles act as a primary iron source, increasing the iron concentration around CSC niches and disrupting redox homeostasis by diminishing glucose-6-phosphate metabolites following lysosomal breakdown and iron metabolism (105). Lie et al. have also devised and fabricated a photoactivated ferrocene-iridium(III) complex (Ir-3) to achieve immunotherapy against melanoma cells, including stem cells. Ir-3 targets mitochondria and disassembles under light irradiation to generate a cytotoxic Ir(III) photosensitizer and Fe2+ ions. These ions produce reactive oxygen species via the Fenton reaction, inducing ferroptosis and autophagy, and ultimately eliciting immunogenic cell death in melanoma cells. Under light exposure, Ir-3 not only induces these effects but also suppresses stem cell-related properties and enhances macrophage-mediated phagocytosis of melanoma stem cells (106).

Overall, combining ferroptosis induction with immunotherapy offers a promising approach to target CSCs and enhance immune responses against cancer. The development of targeted nanotherapies and photoactivated compounds, as demonstrated in these studies, provides a foundation for future therapies that can effectively eliminate CSCs and improve cancer treatment outcomes.

## 6 Stemness and ferroptosis-related biomarkers in cancer

CSCs are pivotal in causing poor survival rates, recurrence, and resistance to therapy in cancer patients (107). Ferroptosis, a form of regulated cell death, plays a crucial role in the survival mechanisms of CSCs (63). Given this, there is a pressing need to identify new biomarkers associated with stemness and ferroptosis to assess their potential in cancer prognosis (**Table 2**). For example, Ruan et al. developed a prognostic model for PDAC based on CSC characteristics and ferroptosis-related genes. This model, which includes genes such as TAGLN2, ARNTL2, ARIH2, FAM83D, MYEF2, WNT7B, ARF6, and TBC1D2, can assist in predicting overall survival and chemotherapy sensitivity in PDAC patients (108). Du et al. identified a set of ferroptosis-related stemness genes (CDKN2A, GABARAPL1, HRAS, RPL8) that may contribute to immune infiltration and prognosis in HCC (97). The m_6_A reader protein IGF2BP3 is negatively correlated with the ferroptosis - related molecule NFE2L2 and positively correlated with SLC1A5 and the immune checkpoint HAVCR2. Additionally, IGF2BP3 shows a positive correlation with the stemness marker SOX2, indicating its potential as a biomarker for predicting ferroptosis and stemness in HCC (109). Dong et al. examined the expression, mutations, and copy number variations (CNVs) of ferroptosis-related genes (FRGs) in glioblastoma and found that these FRGs (NCOA4, TFRC, STEAP3, AKR1C1, and AKR1C3) significantly impact immunity and stemness in glioblastoma (GBM) multiforme, enabling prognosis prediction (110). Similarly, Sui et al. identified nine risk genes in esophageal carcinoma (KLF2, NDRG1, SLC2A1, SLC3A2, STMN1, TIMP1, TNFAIP3, TSC22D3, and TXNIP) and developed the stemness_ferroptosis_index. This index correlates with patients’ clinical traits, with higher scores tending to indicate a worse prognosis (111).

**Table 2:** The significance of stemness and ferroptosis-related biomarkers in cancer.

Signature/Biomarker	Cancer type	Database	Prognostic value	Association	Refs
TAGLN2, ARNTL2, ARIH2, FAM83D, MYEF2, WNT7B, ARF6, TBC1D2	PDAC	TCGA ICGC GEO	OS	correlation with chemotherapy sensitivity	(108)
CDKN2A, GABARAPL1, HRAS, RPL8	HCC	TCGA ICGC	OS	Positive correlation with immune infiltration	(97)
IGF2BP3	HCC	TCGA HPA	OS, PFS	Positive correlation with immune infiltration	(109)
NCOA4, TFRC, STEAP3, AKR1C1, AKR1C3	GBM	GSE13041 TCGA CGGA-325	OS	immune infiltration; ICB responsiveness	(110)
KLF2, NDRG1, SLC2A1, SLC3A2, STMN1, TIMP1, TNFAIP3, TSC22D3, TXNIP	ESCA	TCGA GSE53625	OS	correlated with the clinical characteristics and chemotherapy sensitivity	(111)

TCGA, The Cancer Genome Atlas; ICGC, International Cancer Genome Consortium; GEO, Expression Omnibus; HPA, Human Protein Atlas; OS, overall survival; PFS, progression free survival; ICB, immune-checkpoint blockade.

Overall, the exploration of biomarkers associated with stemness and ferroptosis is of paramount importance and holds the potential to significantly advance cancer prognosis and treatment strategies. Identifying and validating these biomarkers could pave the way for the development of more precise prognostic models and personalized therapeutic approaches. Targeting these biomarkers may also provide innovative therapeutic avenues to overcome resistance to therapy and enhance patient outcomes. As our comprehension of the intricate relationship between stemness and ferroptosis continues to deepen, the field of cancer treatment stands on the threshold of substantial advancements. These progressions are anticipated to lead to improved survival rates and an enhanced quality of life for individuals afflicted with cancer.

## 7 Pharmacological inhibition of ferroptosis and cancer cell stemness

Within cancer treatment, pharmacologically inhibiting ferroptosis and cancer cell stemness symbolizes hope. Various drugs or inhibitors, such as Traditional Chinese Medicine (TCM) and Natural products, have shown potential in targeting cancer cell stemness and inducing ferroptosis (**Table 3**).

**Table 3:** The drugs traget ferroptosis and CSCs in cancer.

Drugs	Cancer	Mechanism	Tumor model	Outcome	Refs.
JYQHD	GC	Inhibition of COL12A1-mediated ROS accumulation	MKN45/ SGC-7901 xenograft	inhibit malignant behaviors	(112)
Tanshinone IIA	GC	Increased the level of lipid peroxides and decreased glutathione level	/	Induce ferroptosis decreased GC stemness	(113)
Ursolic acid	TNBC	Inhibition of KEAP1/NRF2 pathway	MDA−MB−231 xenograft	Induce ferroptosis and inhibit proliferation of CSCs	(114)
CEP	Lung cancer	Inhibition of NRF2 for robust ER stress	Lewis xenograft	Induce ferroptosis and inhibit stemness of lung cancer cells	(115)
Itraconazole	NPC	Increase iron concentration and lipid peroxides oxygen, decrease GSH level	FaDu xenograft	Kill NPC stem cells and thus attenuate radioresistance.	(116)
QPAs	CRC	Induce SLC7A11-mediated ferroptosis	HCT-8 xenograft	Inhibit the stemness of drug-resistant CRC cells	(117)
Butyrate	Lung cancer	Lysosome Fe2+- and SLC7A11-mediated ferroptosis	A549 xenograft	Attenuate the stemness of lung cancer cells	(118)
ATO	Lung cancer	Inhibit m6A modification to promote ferroptosis	LASCs xenograft	Suppresse lung adenocarcinoma stem cell stemness	(119)
Ropivacaine	Ovarian cancer	Inactivate the PI3K/AKT axis	SKOV3 xenograft	Restraine ovarian cancer cell stemness	(120)
Phenazine derivatives	Breast cancer	Accumulate and sequestering iron in lysosomes	MCF-7/MDA-MB-231 xenograft	Inhibit the stemness of breast cancer cells	(121)
NVB	GC	Target POLQ	MGC-803 xenograft	Attenuate the stemness of GC cells and increase ferroptosis sensitivity	(122)
ML-SI1	Breast cancer	Target TRPML1	HCC1954 xenograft	Promote ferroptosis in breast CSCs	(123)

JYQHD, Jian Yun Qing Hua Decoction.

Traditional Chinese Medicine (TCM) is widely used for cancer treatment in Eastern countries (124). Jian Yun Qing Hua Decoction (JYQHD), a TCM prescription, can treat GC by inhibiting cancer cell stemness through the COL12A1-mediated ferroptosis pathway (112). Similarly, Tanshinone IIA, a bioactive compound from the Chinese herb Salvia Miltiorrhiza (Danshen), suppresses cellular stemness in GC cells. It reduces spheroid formation and modulates stemness markers (OCT3/4, ALDH1A1, CD44) by inducing ferroptosis (113). Ursolic acid (UA), a pentacyclic triterpenoid found in various fruits, spices, and medicinal plants, reduces the stemness and proliferation of breast CSCs in spheroid and mouse models by promoting ferroptosis (114). In a similar vein, Cepharanthine (CEP), isolated from Stephania cepharantha Hayata, drives ferroptosis by inhibiting NRF2. This action induces strong ER stress, leading to apoptosis and reduced stemness of lung cancer cells (115). Lastly, Itraconazole, a triazole antifungal drug, can also trigger ferroptosis and diminish the stemness of nasopharyngeal carcinoma (NPC) spheroids (116). Li et al. showed that quinoa-derived polyunsaturated fatty acids (QPAs) can downregulate CD44 expression to suppress the stemness of drug - resistant CRC cells, which inhibits SLC7A11 expression and triggers ferroptosis (117). In lung cancer cells, butyrate, a short-chain fatty acid, recruits lysosomal Fe²^+^ and promoting the ubiquitination-proteasomal degradation of SLC7A11 protein, butyrate induces ferroptosis in lung CSCs (118).

In the field of ancient Chinese medicine, arsenic trioxide (As_2_O_3_, ATO) has been proven to be a low-toxicity yet effective anticancer agent for acute promyelocytic leukemia and certain solid cancers (125). ATO can reduce the stemness of lung adenocarcinoma CSCs by inducing ferroptosis. This is achieved through inhibiting ZC3H13 gene expression, which in turn suppresses m_6_A modification (119). Similarly, ropivacaine, a commonly used local anesthetic, has been found to suppress the growth and stemness of ovarian cancer cells while promoting ferroptosis via the PI3K/AKT signaling pathway (120). Another notable example is phenazine derivatives (CPUL119, CPUL129, CPUL149), which significantly inhibit breast cancer cell stemness by triggering ferroptosis. They work by accumulating iron in lysosomes through iron interaction and by modulating iron - related proteins (IRP2, TfR1, ferritin) (121). In addition, novobiocin (NVB), a Polymerase theta (POLQ) inhibitor undergoing multiple clinical trials (126), could reduce GC cell stemness and increases their ferroptosis sensitivity (122). In breast cancer cells, the lysosomal cation channel TRPML1 inhibitor ML-SI1 can trigger ferroptosis in breast CSCs. It reduces their stemness and increases breast cancer cell sensitivity to the chemotherapeutic agent doxorubicin (123). These substances serve as prime examples of how different compounds can combat cancer by reducing cell stemness and inducing ferroptosis, highlighting their potential for cancer treatment.

In summary, drugs, including TCM, Natural products and inhibitor, show significant advantages in inducing ferroptosis and inhibiting CSCs. Nevertheless, several challenges impede their clinical translation. The regulatory networks linking ferroptosis and stemness, such as those mediated by NRF2, SLC7A11, and iron metabolism genes, require further elucidation to decipher the multi-target mechanisms of these compounds within the tumor microenvironment (TME). Additional limitations include the poorly defined mechanisms of complex agents like JYQHD and QPAs, CSC heterogeneity, TME-mediated modulation of ferroptosis, and poor drug delivery to CSC niches. To address these barriers, efforts are underway to advance clinical translation through the repurposing of agents in clinical trials (e.g., Novobiocin), standardization of herbal preparation protocols, and the development of rational combination therapies such as “ferroptosis inducers + stemness inhibitors + conventional chemotherapy” (e.g., ML-SI1 with doxorubicin), offering a promising strategy to overcome treatment resistance and eradicate CSCs.

## 8 Conclusions and perspectives

**Figure 4:**
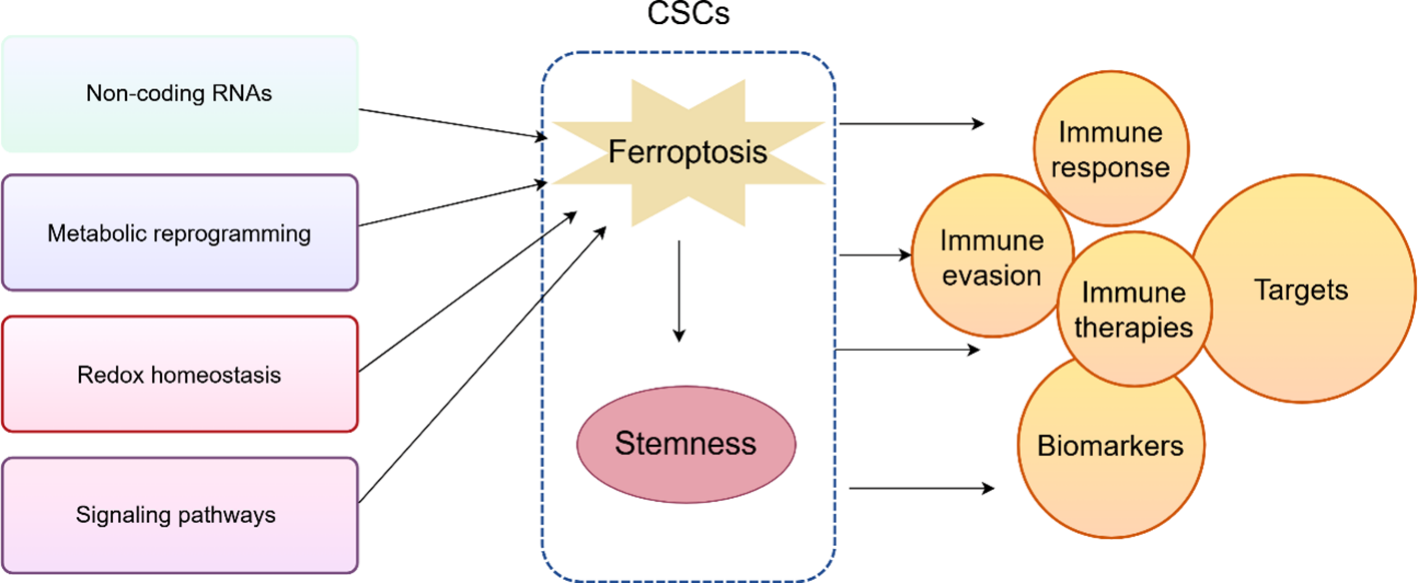
The schematic summary of the interplay between ferroptosis and CSCs. The graphic was created by Figdraw (www.figdraw.com).

The discovery of ferroptosis has expanded the understanding of cell death and revealed novel therapeutic strategies for cancer. We discusses the interplay between ferroptosis and cellular stemness, its impact on the tumor immune microenvironment, and the role of ferroptosis in CSC immune evasion. We also summarize associated biomarkers and pharmacologic approaches to induce ferroptosis in CSCs (**Figure 4**).

Despite the optimism from significant advancements in studying ferroptosis and CSCs, many questions remain unanswered. Major challenges include figuring out the complex molecular interaction mechanisms between them and finding unique biomarkers, particularly *in vivo*. Current research mostly uses *in vitro* and mouse models, which may not reflect human cancer clinical manifestations. Also, both CSCs and non-CSCs are highly plastic and dynamic, changing types in response to environmental stimuli. Moreover, few molecular targets for CSC function intervention have been clinically translated, with high clinical trial failure rates.

Future research should center on mechanism analysis, target mining, clinical transformation, therapeutic strategy development, and transformation path optimization. It’s crucial to deeply explore their interaction mechanisms within the TME, such as ncRNA-regulated epigenetics, metabolic reprogramming, and redox imbalance. This will help map cross-pathway interaction networks and identify “dual - effect targets” like SLC7A11 and NRF2. Simultaneously, efforts should focus on clinical transformation of stemness-ferroptosis related biomarkers. Develop diagnostic tools based on specific molecules for early cancer screening, therapeutic monitoring, and recurrence prediction, and establish biomarker-guided stratification treatment models.

While this review has synthesized evidence across multiple tumor types to establish the core interplay between ferroptosis and CSCs, it is important to note that the specific molecular drivers and the relative contribution of this axis to tumor progression may vary across different cancer contexts. Factors such as tissue-of-origin genetic landscapes, and unique tumor microenvironments can influence both ferroptosis sensitivity and CSC phenotypes. A systematic, comparative analysis of these differences represents a critical frontier for future research, which will be essential for tailoring ferroptosis-based therapies to specific cancer types.

Therapeutically, ferroptosis-based modalities have demonstrated promising efficacy across various cancers, including OSCC (127). Notably, in OSCC cells derived from never-smokers, nuclear receptor coactivator 4 (NCOA4)-mediated ferritinophagy has been implicated as a critical upstream mechanism driving cadmium-induced ferroptosis (128). Therefore, rationally designed dual-pathway inhibitors that concurrently eradicate the cancer-stem-cell (CSC) pool and remodel the tumor micro-environment should be delivered via nano-carriers or CRISPR-based gene-editing platforms. However, targeting ferroptosis in the clinic still faces multiple bottlenecks—namely, sub-optimal drug release kinetics, on-target/off-tumor toxicities (e.g., GPX4-low renal tubules), and the paucity of validated ferroptosis-inducing agents that have advanced beyond early-phase trials. Addressing these obstacles will be critical to overcoming the ferroptosis-evasion-mediated drug resistance exhibited by CSCs. Efforts should therefore broaden the applicability of such combination regimens across diverse cancer histologies, leverage multi-omics data, next-generation pre-clinical models and artificial intelligence to accelerate the closed-loop translation from “mechanism → biomarker → therapy”.

In conclusion, the review emphasize the connection between ferroptosis and CSCs in cancer, making them promising biomarkers and therapeutic targets. The progress in the field will depend on interdisciplinary collaboration, technological innovation, and an enhanced understanding of ferroptosis and cancer cell stemness. These efforts will yield new disease - mechanism insights and reveal novel therapeutic - intervention avenues.
